# Effectiveness of endoscopic ultrasound-guided drainage procedure for symptomatic lymphocele

**DOI:** 10.1055/a-2072-3732

**Published:** 2023-05-04

**Authors:** Koichi Soga, Shun Takakura, Akinobu Sai

**Affiliations:** Department of Gastroenterology, Omihachiman Community Medical Center, Omihachiman, Shiga, Japan

A 65-year-old Japanese woman was admitted to our hospital with complaints of abdominal pain and a fever of 39.0 °C. Computed tomography (CT) revealed an infected lymphocele after surgery for ovarian cancer. Endoscopic ultrasound-guided lymphocele drainage (EUS-LD) was planned while avoiding free space between the colon and lymphocele using a convex EUS scope ( Fig. 1 ).

**Fig. 1:**
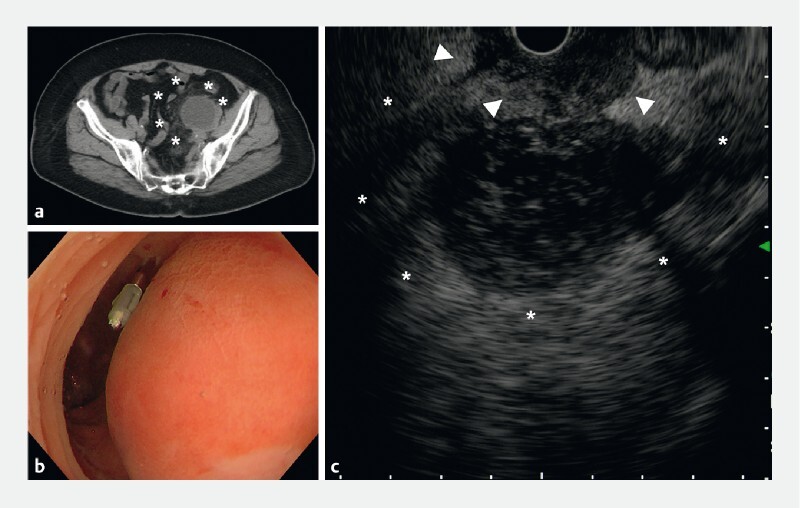
Computed tomography revealed an infected lymphocele after an operation for ovarian cancer. **a** The lymphocele (asterisk) was near the sigmoid colon and appeared partially adherent to the sigmoid colon. **b** A colonoscopy revealed erythematous tone and mucosal elevation that appeared to be a spillover of inflammation from an infected lymphocele. In the miniature probe and convex endoscopic ultrasound (EUS) procedure, after aspirating the residual colonic air, VISCOCLEAR gel was injected through the accessory channel to secure the visual field without gas insufflation. **c** EUS showed an infected lymphocele (asterisk) and inflammatory spillover (arrowhead) into the surrounding area, which was adherent to the colon.

During the EUS procedure, VISCOCLEAR gel (Otsuka Pharmaceutical Factory, Tokushima, Japan) was injected through the accessory channel to secure the visual field without gas insufflation 1 . EUS showed an inflammatory spillover connected to the lymphocele adherent to the sigmoid colon as the puncture route. The lymphocele was punctured using a 19-gauge needle (EZ Shot 3 Plus; Olympus, Tokyo, Japan). After aspirating the lymphocele contents, a guidewire was inserted through the needle and coiled into the abscess cavity. Because of the narrow and short space, we anticipated an easy shift in the endoscope’s position. To ensure it remained stable during the procedure, we used novel drill dilator devices (Tornus ES; Olympus, Tokyo, Japan) to avoid applying unnecessary pushing force 2 3 4 . Thereafter, a double-pigtail plastic stent (7-Fr, 7 cm long) (Through & Pass; Gadelius, Tokyo, Japan) was placed into the lymphocele. This procedure was performed safely without complications and induced a good clinical course ( Fig. 2 , Video 1 ).

**Fig. 2:**
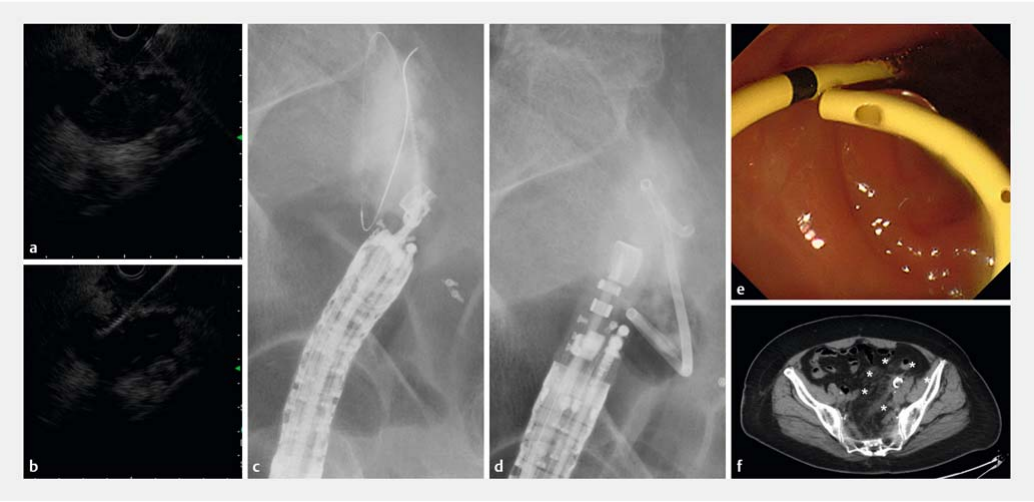
Endoscopic ultrasound-guided lymphocele drainage (EUS-LD) was planned using a convex EUS scope. **a** The abscess was punctured using a 19-gauge needle via the sigmoid colon. **b, c** Since the endoscopy was performed in a narrow and short space, we anticipated it could readily shift its position. We used novel drill dilator devices to avoid applying unnecessary pushing force to keep the endoscope stable. **d** Subsequently, a double-pigtail plastic stent (DPPS; 7-Fr, 7 cm long) was placed into the lymphocele. **e** After the DPPS insertion, infectious matter started draining into the colon. **f** Abdominal computed tomography after the procedure revealed the correct placement of the drainage tube in the lymphatic cyst and completely drained cyst.

**Video 1** An effective procedure for treating lymphocele using endoscopic ultrasound-guided lymphocele drainage.


A lymphocele is a cystic mass that may occur in the retroperitoneum following a systematic pelvic and/or para-aortic lymphadenectomy. The most severe complication of a lymphocele is infection. Generally, minimally invasive methods involving catheter drainage and sclerotization tend to be popular; however, surgery remains the first option in recurring, poorly accessible or inflammatory lymphoceles 5 . We believe that EUS-LD can help effectively control an infected lymphocele. Notably, the gel immersion method and drill dilator devices can help to perform the procedure more safely and efficiently.

Endoscopy_UCTN_Code_TTT_1AS_2AG

## Notes

Endoscopy E-Videos

https://eref.thieme.de/e-videos
*E-Videos* is an open access online section of the journal *Endoscopy* , reporting on interesting cases and new techniques in gastroenterological endoscopy. All papers include a high-quality video and are published with a Creative Commons CC-BY license. Endoscopy E-Videos qualify for HINARI discounts and waivers and eligibility is automatically checked during the submission process. We grant 100% waivers to articles whose corresponding authors are based in Group A countries and 50% waivers to those who are based in Group B countries as classified by Research4Life (see: https://www.research4life.org/access/eligibility/ ). This section has its own submission website at https://mc.manuscriptcentral.com/e-videos
